# Application of a warfarin dosing calculator to guide individualized dosing *versus* empirical adjustment after fixed dosing: a pilot study

**DOI:** 10.3389/fphar.2023.1235331

**Published:** 2023-08-17

**Authors:** Xiaofang Cai, Jiana Chen, Maohua Chen, Xiaotong Xia, Guanhua Fang, Jinhua Zhang

**Affiliations:** ^1^ Department of Pharmacy, Fujian Maternity and Child Health Hospital College of Clinical Medicine for Obstetrics and Gynecology and Pediatrics, Fujian Medical University, Fuzhou, China; ^2^ Department of Pharmacy, Fujian Medical University Union Hospital, Fuzhou, China; ^3^ Zhangpu County Hospital, Zhangzhou, China; ^4^ Pingtan Comprehensive Experimental Area Hospital, Pingtan Comprehensive Experimental Area, Fuzhou, China; ^5^ Department of Cardiac Surgery, Union Hospital, Fujian Medical University, Fuzhou, China

**Keywords:** thrombus, dose calculator, warfain, INR, TTR, bleeding

## Abstract

**Background:** Warfarin has a narrow therapeutic window and individual variation, and patients require regular follow-up and monitoring of the International Normalized Ratio (INR) for dose adjustment. The calculation method of Warfarin Dosing Calculator (WDC) software is based on the European and American populations, and its accuracy in the Chinese population is yet to be verified.

**Objective:** This study was to evaluate the feasibility of applying Warfarin Dosing Calculator software intervention in a real-world clinical research setting in China.

**Methods:** The pilot study divided the included patients after valve replacement into an experimental group and a control group, with 38 cases in each group. In the control group, the initial dose was fixed at 2.5 mg/d and the dose was adjusted empirically during the study period; in the experimental group, the Warfarin Dosing Calculator software was applied to guide the dosing, and patients in both groups were followed up for 3 months. Analysis of the incidence anticoagulation outcomes and excessive anticoagulation events in both groups. Kaplan-Meier survival curves were used to analyze the correlation between different dosing regimens and first International Normalized Ratio attainment, and Logrank tests were performed.

**Results:** The mean time required for first International Normalized Ratio compliance in the experimental group was 4.38 days less than in the control group, and the mean number of tests was 1.43 less (*p* < 05). Time in therapeutic range (TTR) was significantly higher in the experimental group than in the control group (*p* < 05). Kaplan-Meier survival curve analysis showed that the first International Normalized Ratio attainment rate was significantly higher in the experimental group than in the control group (*p* = 01). No major bleeding events occurred in either group, but other excessive anticoagulation events (INR>3.5 and minor bleeding) were significantly reduced in the experimental group compared with the control group (*p* < 05).

**Conclusion:** Application of Warfarin Dosing Calculator software to guide individualized warfarin dosing may be better than a fixed dose of 2.5 mg/d. It may be shorten the time to first International Normalized Ratio attainment, and the attainment rate in the same time, and can better improve the mean Time in therapeutic range level value and reduce excessive anticoagulation events, which improves the safety of warfarin anticoagulation therapy in clinical practice.

**Clinical Trial Registration:** https://www.chictr.org.cn/showproj.html?proj=52793, ChiCTR2000032393.

## Introduction

Warfarin is an oral anticoagulant of the coumarin class, which exerts its anticoagulant effect by inhibiting vitamin K. It is mainly used in the treatment of deep vein thrombosis and pulmonary embolism and in the prevention of thrombosis in cardiac valve disease, mechanical valve replacement and atrial fibrillation (Kearon et al., 2016; Vahanian et al., 2022). However, warfarin has a narrow therapeutic window and individual variation, and patients require regular follow-up and monitoring of the International Normalized Ratio (INR) for dose adjustment. Current basic methods for constructing warfarin dosing models include multiple linear regression analysis and nonlinear mixed effects modeling (NONMEM) methods (Lin et al., 2014; Tan et al., 2015), and NONMEM method is considered the “gold standard” for drug modeling (Sennesael et al., 2018).

The area of Mobile health (Mhealth) continues to grow globally: in June 2021, there were over 350,000 health-related mobile apps worldwide, with more than 250 new apps being added to web-based stores every day. (Digital Health Trends, 2021, 2021). Mobile healthcare brings convenience to people and promotes the development of medicine. (Cao et al., 2021). Due to the complexity of warfarin dose adjustment, mobile medicine related to warfarin dose adjustment has also emerged. Hamberg applied the warfarin PK/PD model constructed by NONMEM software to a warfarin dosing calculator (WDC) written in JAVA for children and adults. The software is not only simple and convenient to use, but also can predict the dosage of warfarin, the efficacy after administration and the adverse reactions after inputting the patient’s clinical data (Hamberg et al., 2015). The WDC software is under additionalfiles after the paper of Hamberg et al. (Hamberg et al., 2015) and the instructions for its use are attached to this paper.

There was a significant difference in the stable dose of warfarin between races, with a mean stable dose of 5.7 mg/d for black people, 4.5 mg/d for white people, and 2.5–3.0 mg/d for Asian people (Lenzini et al., 2010). However, the calculation method of WDC software is established according to the European and American population, and its accuracy has not been verified in the Chinese population. Therefore, this study will explore the use of WDC software to predict warfarin dose and fixed administration of 2.5 mg/d with empirical dose adjustment in a Chinese population, and compare the significance of these two administration methods for the anticoagulant guidance of warfarin in patients after heart valve replacement.

## Materials and methods

### Trial design

This study is an open-label pilot study comparing predicted warfarin dose using WDC software with empirical adjustment after fixed 2.5 mg/d administration. The patients included after heart valve replacement were divided 1:1 into experimental and control groups. The target sample size was determined based on available resources and the practicality of recruitment and assessment. And the rate of viable outcomes (e.g., retention rate) was estimated with a standard error of no greater than 0.1. This study has been registered (ChiCTR2000032393).

Inclusion criteria: i) age ≥18 years; ii) patients after valve replacement; iii) regular warfarin administration (regular daily dose, no missed dose); iv) patients and families were clear about the study objectives and procedures and were voluntary. Exclusion criteria: i) malignant tumor; ii) severe mental illness or psychiatric disorder; iii) pregnancy or lactation; iv) severe renal insufficiency (endogenous creatinine clearance <15 mL/min and serum creatinine ≥200 μmol/L (Motykie et al., 1999)); v) Severe hepatic insufficiency (bilirubin 2 times higher than the upper limit of normal, and Aspartate Transaminase (AST)/Alanine Aminotransferase (ALT)/Alkaline Phosphatase 3 times higher than the upper limit of normal) (Wright and Duffull, 2013); vi) CYP2C9 and VKORC1 gene test results were lacking.

Participants were enrolled between September 2020 and December 2020 in Fujian Medical University Union Hospital. All enrolled patients signed an informed consent form, and the study was approved by the Ethics Committee of Union Medical College Hospital, Fujian Medical University (2020KY006). Relevant demographic information was collected, including age, sex, height, weight, and any combination of amiodarone, statins, broad-spectrum antimicrobials, and azole antifungals. Co-administered drugs are drugs that patients take for more than 3 consecutive days before reaching a stable dose of warfarin, which are reported in the literature to have an enhancing or inhibiting effect on warfarin (Ansell et al., 2008).

### INR target range

The INR target ranges for each anti-coagulation indication in this study are shown in Table 1. If the patient has a combination of multiple anti-coagulation indications, a higher INR target range corresponding to the indications will be used in the development of the dose adjustment protocol. It is worth noting that the target range of INR after heart valve surgery is derived from the clinical experience of cardiovascular surgery at the main central hospital, and therefore is not entirely consistent with the target range recommended by the Chinese Guidelines for the Prevention and Treatment of Thrombotic Diseases. This INR target range has been applied to more than 40,000 patients for more than 30 years and can ensure the security and efficacy of anti-coagulation therapy.

**TABLE 1:** INR target ranges for different indications.

Anticoagulation indication	INR target range
Aortic Valve Replacement or Repair	1.5–2.0
Mitral Valve Replacement or Repair	1.7–2.5
Tricuspid Valve Replacement or Repair	2.0–2.5
Atrial Fibrillation	2.0–3.0
Venous Thromboembolism	2.0–3.0

### INR monitoring frequency

The frequency of initial INR testing for patients after discharge from hospital is once a week. When two consecutive INR values are within the patient’s target range, the patient’s INR testing interval may be extended by 1 week, i.e., once every fortnight. In the case of fortnightly INR values, if two consecutive INR values are within the patient’s target range, the blood check interval can be extended by another week, i.e., once every 3 weeks, and so on. The maximum interval between blood tests should not exceed 1 month (INR values need to be tested at least once a month). If the warfarin dose needs to be adjusted, revert to weekly blood checks and repeat the process until the dose is stable again.

### Dose adjustment scheme

In the experimental group, WDC software was applied to guide the dosing, and the basic information of patients and genetic test results were brought into the software to predict the initial dose. As the commonly used warfarin dose in China is 2.5 mg/tablet *versus* 3 mg/tablet, the minimum separated dose is 0.625 mg, and the dose is given according to the principle of proximity. (Liu et al., 2021). During the experiment, if a patient’s tested INR value exceeds the target range, the trend of the next INR value is predicted based on the previously tested INR and medication use brought into the WDC software to evaluate whether to adjust the dosing regimen.

The initial dose of warfarin in the control group was 2.5 mg/d. During the experiment, dose adjustment was required if the patients’ tested INR values exceeded the target range. The dose adjustment protocol for warfarin is empirically developed by clinical pharmacists or physicians based on information such as INR results, co-morbidities and medications, and clinical events with reference to the Chinese Expert Consensus on Warfarin Anti-coagulation Therapy. The general principles of dose adjustment are as follows:

1) If the INR value is within ±0.2 of the upper and lower limits of the target range, the warfarin dose remains unchanged.
2) If INR < lower limit of target range −0.2, warfarin increases by 0.625 mg.
3) If upper limit of target range +0.2 < INR ≤3, warfarin decreases by 0.625 mg.
4) If INR>3, stop the drug for 1 day and recheck INR on the next day. If INR≤3.0 on the next day, reduce 0.625 mg and repeat the above process; if INR>3.0 on the next day, continue to stop the drug until INR≤3.0.

### Outcome indicators

The primary outcome indicator in this study was Time in therapeutic range (TTR), and the secondary outcome indicators were the occurrence of clinical events (safety and effectiveness outcomes) and the distribution of INR.

### Safety outcomes

Over-anticoagulation events that occurred during the study period were counted for both groups of patients. Over-anticoagulated events include major bleeding events, minor bleeding events and INR >3.5 events as defined by the International Society of Thrombosis and Hemostasis (ISTH) (Schulman et al., 2005; Schulman et al., 2010). Where major bleeding is defined as meeting any of the following: i) fatal bleeding, and/or; ii) bleeding in a critical area or organ, such as intracranial, intraspinal, intraocular, retroperitoneal, intra-articular or pericardial, or intramuscular with compartment syndrome, and/or; iii) bleeding causing a fall in hemoglobin level of 20 g/L^−1^ (1.24 mmol/L^−1^) or more, or leading to transfusion of two or more units of whole blood or red cells. Minor bleeding events are those that include gingival bleeding, non-traumatic skin mucosal petechiae, nasal bleeding, occult urinary bleeding, etc. And any other clinical bleeding that does not meet the criteria for major bleeding.

### Effectiveness outcomes

The anticoagulation results during the study period were counted for both groups of patients. The following aspects were included: i) the occurrence of thromboembolic events; ii) the time required to reach the target INR value for the first time and the number of tests; and iii) TTR during hospitalization, follow-up at 1 month and 3 months in both groups. The TTR in this study was based on an algorithm of the percentage of days to standard, combining the changes in the INR values of the patients several times after the administration of the drug to derive the percentage of their possible days to standard INR during anticoagulation therapy (Chinese Society of Cardiovascular Diseases and Cardiovascular and Cerebrovascular Diseases Committee of the Chinese Society of Gerontology, 2013).

### Statistical analysis

SPSS 25 software was applied for statistical analysis. Count data in the results were expressed as frequencies and percentages, and the χ^2^ test was used for comparison between groups; measurement data were expressed as mean ± standard errors, and the *t*-test was used for comparison between groups. Kaplan-Meier survival curves were used to analyze the correlation between different dosing regimens and the endpoint event (first attainment of INR), and Logrank test was performed, and the difference was considered statistically significant at *p* < .05.

## Results

### Basic information

A total of 96 participated were eligible and were approached during the trial period. 16 participated were eligible but could not be recruited. Of these, 11 participated declined to participate due to technical difficulties or refusal to follow up on treatment, and 5 participated in other studies. Of the 82 participated included, 4 (5%) participated dropped out midway through the study and the dropout rate was low, resulting in 76 valid participants, 38 participated each in the experimental and control groups. Patients in the experimental group had a mean age of 54.74 ± 2.01 years, a mean weight of 59.28 ± 1.36 kg, and a mean height of 163.763 ± 1.256 cm; patients in the control group had a total mean age of 50.00 ± 1.81 years, a mean weight of 59.28 ± 1.36 kg, and a mean height of 163.447 ± 1.219 cm. None of the patients’ basic information was statistically significant when compared between groups (*p* > .05), as detailed in Table 2.

**TABLE 2:** Basic clinical data of the patients.

		Experimental group (N = 38)		Control group (N = 38)	*p*-Value
**Basic Information**					
Age (year)		54.737 ± 2.011		50.000 ± 1.805	.08
Height (cm)		163.763 ± 1.256		163.447 ± 1.219	.86
Weight (kg)		59.276 ± 1.356		59.276 ± 1.356	.89
Sex (female)		17 (0.447)		15 (0.395)	1.00
CYP2C9 *1*3		1 (0.026)		4 (0.105)	.15
CYP2C9 *1*1		37 (0.974)		34 (0.895)	.15
*VKORC1 GA/GG*		3 (0.079)		7 (0.184)	.17
*VKORC1 AA*		35 (0.921)		31 (0.816)	.17
**Risk factors**					
Basic INR^a^		1.001 ± 0.014		1.032 ± 0.012	.17
Smoking		14 (0.368)		11 (0.289)	.46
Drinking		13 (0.342)		14 (0.368)	.81
Diabetes		7 (0.184)		9 (0.237)	.57
Hypertension		10 (0.263)		8 (0.211)	.59
**Purpose and dosage of medication**					
Aortic valve		21 (0.553)		18 (0.474)	.49
Mitral valves		17 (0.447)		20 (0.526)	.49
Achieved dose (mg/d)		3.242 ± 0.161		3.39 ± 0.213	.60
**Combination of medications**					
Broad-spectrum antibacterial drugs		3 (0.079)		4 (0.105)	.69
Amiodarone		8 (0.211)		3 (0.079)	.10
Azole antifungal drugs		1 (0.026)		2 (0.053)	.25
Statins		4 (0.105)		2 (0.053)	.39

^a^ INR: international normalized ratio.

### Clinical outcomes

#### Safety outcomes

The number of events with INR>3.5 was 3 cases (8%) in the experimental group and 10 cases (26%) in the control group, with a statistically significant difference between the two groups (*p* = .03). The number of events with minor bleeding was 2 cases (5%) in the experimental group and 8 cases (18%) in the control group, and the difference between the two groups was statistically significant (*p* = .04), see Table 3 for details.

**TABLE 3:** Comparison of excessive anticoagulation events in the two groups.

	Experimental group (N = 38)	Control group (N = 38)	*p*-Value
INR^a^>3.5	3 (0.079)	10 (0.263)	.03
Minor bleeding	2 (0.053)	8 (0.184)	.04

^a^ INR: international normalized ratio.

#### Effectiveness outcomes

**FIGURE 1:**
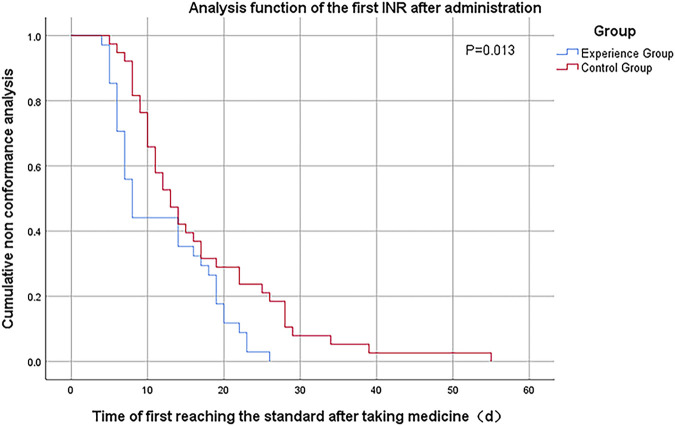
Kaplan-Meier analysis curve for the first INR standard after medication.

No thrombotic events in either group. The mean time to the first INR compliance time was 7.550 ± 0.749 days in the experimental group and 11.158 ± 1.271 days in the control group, with a statistically significant difference between the two groups (*p* = .02). Total number of tests for first INR compliance was 3.263 ± 0.274 in the experimental group and 4.842 ± 0.428 in the control group, and the difference between the two groups was statistically significant (*p* = .004). The TTR during hospitalization was 33.208 ± 2.494 in the experimental group and 24.057 ± 2.412 in the control group, with a statistically significant difference between the two groups (*p* = 0.010). The TTR at 1-month follow-up was 68.634 ± 2.259 in the experimental group and 49.574 ± 2.849 in the control group, and the difference between the two groups was statistically significant (*p* < .01). The TTR at 3-month follow-up was 77.763 ± 1.773 in the experimental group and 57.429 ± 2.656 in the control group, and the difference between the two groups was statistically significant (*p* < .01), as detailed in Table 4. Kaplan-Meier survival curves for both groups of patients were analyzed for correlation between different dosing regimens and first INR value attainment with Logrank test, showing a statistically significant difference (*p* = .01), as detailed in Figure 1.

**TABLE 4:** Comparison of the anticoagulation results between the two patient groups.

Anticoagulation results	Experimental group (N = 38)	Control group (N = 38)	*P*
First INR^a^ compliance time (day)	7.550 ± 0.749	11.158 ± 1.271	.02
Total number of tests for first INR compliance	3.263 ± 0.274	4.842 ± 0.428	.004
TTR^b^ during hospitalization	33.208 ± 2.494	24.057 ± 2.412	.01
TTR at 1-month follow-up	68.634 ± 2.259	49.574 ± 2.849	<0.001
TTR at 3-month follow-up	77.763 ± 1.773	57.429 ± 2.656	<0.001

^a^ INR: international normalized ratio.

^b^ TTR: time in therapeutic range.

## Discussion

Although domestic and foreign researchers have developed many warfarin models with clinical application value, most of the models remain at the level of “model construction”. The application of modeling and simulation technology to develop accurate warfarin dosing regimens for patients is the ultimate goal of model-guided warfarin precision medication. This critical step is the development and application of the clinical decision supporting system (CDSS). At present, publicly available CDSS is mainly based on multiple linear regression analysis and population PK/PD models, and is mainly based on computer platforms, web platforms, and mobile devices. Such as WDC, WarfarinDosing, iWarfarin software, etc. (Zhang et al., 2022)

In our study, the mean time required for first INR compliance in the experimental group was 4.38 days less than in the control group, and the mean number of tests was 1.43 less (*p* < .05). Time in therapeutic range (TTR) was significantly higher in the experimental group than in the control group during study (*p* < .05). Kaplan-Meier survival curve analysis showed that the first INR attainment rate was significantly higher in the experimental group than in the control group (*p* = .01). No major bleeding events in either group. However, other excessive anticoagulation events (INR>3.5 and minor bleeding) were significantly reduced in the experimental group compared with the control group (*p* < .05).

The time required to reach the target INR value for the first time in the experimental group in this study was less than that in the control group (*p* = .02), similar to the findings of Marek E (Marek et al., 2016) and Dong J (Dong et al., 2019) et al. Both indicated that the use of WDC software can better predict the initial dose of warfarin and shorten the time to reach the target INR value. However, none of them were compared for TTR value improvement, while this study was further investigated and found that the overall TTR values of the patients in the experimental group were all improved compared to the control during the follow-up period. Not only did they improve the overall time patients spent at the target value, but they also improved the safety of the patients on the medication. In another study (Al-Metwali et al., 2019), the WDC group took less time to reach a stable INR than the control group (29.0 vs. 96.5 days), but took more time to first reach a standard INR value than the control group (5 vs. 2 days), which was different from our results. The inclusion population of that study was non-Asian children, and ethnic and age differences may have contributed to the different results from our study.

The TTR was lower in both groups during hospitalization, mainly because the INR was still unstable at the beginning of the patients’ dosing and the slow onset of warfarin made the attainment rate lower (Holford, 1986). The TTR value increased significantly with the prolongation of drug administration, with the TTR value of the experimental group exceeding 68.63% at 1 month of follow-up and reaching 77.76% at 3 months. In contrast, the TTR of the control group was only 49.57% at 1 month and 57.43% at 3 months. This result is similar to a study in a Chinese population, which showed that clinicians empirically adjusted warfarin doses based on INR values only, with TTR values in the range of 40%–65% (Dong et al., 2019). In contrast, it is usually considered that patients taking warfarin should have a TTR value greater than 65% to ensure a better anticoagulant effect of warfarin with fewer bleeding adverse effects. This indicates that individualized warfarin dosing guided by the WDC software is more accurate and effective than empirical adjustment of warfarin dose based on INR values alone. There may be several reasons for the improved TTR in the experimental group. First, patients in the experimental group may have received more detailed dosing instructions from the pharmacist, and patients may have been more compliant and more able to review regularly. Secondly, the application of WDC software can predict the INR value before the next follow-up and can make better dose adjustment.

Kaplan-Meier survival curves showed that patients started to achieve INR values on day 5, and the cumulative achievement rate in the experimental group became higher and significantly higher than that in the control group as time progressed. The cumulative attainment rate was not significantly different between the two groups from day 14 to day 19, which may be due to the delay in attaining a new INR steady state in the patients of the experimental group who needed a change in the starting dose. And after day 19, the cumulative attainment rate of the experimental group was again significantly higher than that of the control group, and by day 26, all patients in the experimental group had attained the standard, while the cumulative attainment rate of the control group was only (56.79 ± 0.80)%. Overall comparison, the first INR attainment rate was significantly higher in the experimental group than in the control group at the same time (*p* = .01). The reason for this is that the half-life of warfarin is 36–42 h, and factors II, IX, and X are depleted after about 3 days of administration to show the full anticoagulant effect, and the efficacy is stable after 5–7 days of administration (Hirsh et al., 1995).

In terms of adverse reactions, although no major bleeding or thromboembolic events occurred in either group during follow-up, the incidence of minor bleeding was 18.4% (8 cases) in the control group, which was significantly higher than that of 5.3% (2 cases) in the experimental group. The lower TTR may be the reason for the higher number of bleeding in the control group. Tavares LC et al. (Tavares et al., 2018) and Amin A et al. (Amin et al., 2014) studies also showed that patients with higher TTR had lower rates of adverse events such as bleeding and thromboembolism, and that patients were more likely to have bleeding or embolic events when the TTR was ≤60%. Also, the incidence of INR>3.5 in the experimental group was 7 cases less than that in the control group, and all the differences were statistically significant. Thus, using the WDC software may reduce the incidence of side effects and delay the occurrence of adverse reactions, resulting in a safer clinical application of warfarin. In another study (Jiang et al., 2018) that validated the clinical value of the Lou-type model with a Chinese population, the results showed that the experimental group reached a stable dose in a greater number of cases and took less time to reach a stable dose compared to the control group, which is consistent with our findings. However, the incidence of adverse reactions in that study was lower than in our study, probably because the follow-up period in that study was only 50 days, whereas ours was 3 months, and some adverse reactions may have been missed because the follow-up period was too short. Therefore, an appropriately longer follow-up period may help to obtain more realistic data.

The current study is presented as a pilot study, the purpose of which is to evaluate the feasibility of applying this software intervention in a real-world clinical study setting. Afterwards, we will build on this study to comprehensively assess the influence of genetic and non-genetic factors on the pharmacokinetic and pharmacodynamic characteristics of a population-based warfarin PPK/PD model in nine study centers in China (distributed in eastern, central, southern, southwestern and northwestern China) based on population pharmacokinetic and pharmacodynamic characteristics of multi-center Chinese populations in different regions of the country. Based on the model constructed by Hamberg, we will use the NONMEM method to construct a warfarin PPK/PD model based on the Chinese population.

The present study has some novelty features. First, this study is the first to apply WDC software to prospectively predict the initial dose of patients in a Chinese population. Secondly, in the relevant data reviewed so far, except for the study by Wright DF et al. (Wright and Duffull, 2013), for most prospective studies, only the prediction of the initial dose or stable predicted dose was done using the WDC software. In contrast, our study further predicted INR values and doses for patients at different times and states, as well as follow-up statistics for TTR.

There are some limitations of this study. First, participants in this study were not randomly assigned to the experimental and control group groups, which may be subject to selection bias and other potential confounding variables. Second, we did not study antiplatelet drugs such as aspirin, which could potentially affect the pharmacokinetic effects of warfarin. Third, the sample size of this study was small (76 participants) and from the same center, which may have affected statistical power. Therefore, the study findings could be further validated by expanding the sample size in multiple centers in the future.

## Conclusion

The application of WDC software to guide individualized warfarin dosing may be better than the initial dose of fixed 2.5 mg/d, which can shorten the time to first INR compliance, the number of tests and the INR compliance rate, and can better improve the mean TTR level value and reduce excessive anticoagulation events, which has potential significance for clinical improvement of the safety and efficacy of warfarin anticoagulation therapy.

## Data Availability

The original contributions presented in the study are included in the article/supplementary material, further inquiries can be directed to the corresponding author.

## References

[B1] Al-Metwali B. Z. Rivers P. Goodyer L. O'Hare L. Young S. Mulla H. (2019). Personalised warfarin dosing in children post-cardiac surgery. Pediatr. Cardiol. 40 (8), 1735–1744. 10.1007/s00246-019-02215-y 31587090 PMC6848240

[B2] Amin A. Deitelzweig S. Jing Y. Makenbaeva D. Wiederkehr D. Lin J. (2014). Estimation of the impact of warfarin's time-in-therapeutic range on stroke and major bleeding rates and its influence on the medical cost avoidance associated with novel oral anticoagulant use-learnings from ARISTOTLE, ROCKET-AF, and RE-LY trials. J. Thromb. Thrombolysis 38 (2), 150–159. 10.1007/s11239-013-1048-z 24477787

[B3] Ansell J. Hirsh J. Hylek E. Jacobson A. Crowther M. Palareti G. (2008). Pharmacology and management of the vitamin K antagonists: american College of chest physicians evidence-based clinical practice Guidelines (8th edition). Chest 133 (6), 160S-198S–198S. 10.1378/chest.08-0670 18574265

[B4] Cao H. Jiang S. Lv M. Wu T. Chen W. Zhang J. (2021). Effectiveness of the alfalfa app in warfarin therapy management for patients undergoing venous thrombosis prevention and treatment: cohort study. JMIR Mhealth Uhealth 9 (3), e23332. 10.2196/23332 33650976 PMC7967226

[B5] Chinese Society of Cardiovascular Diseases, Cardiovascular and Cerebrovascular Diseases Committee of the Chinese Society of Gerontology (2013). Chinese expert consensus on warfarin anticoagulation therapy. Chin. J. Intern. Med. [J] 52 (001), 76–82. 10.3760/cma.j.issn.0578-1426.2013.01.027

[B6] Digital Health Trends 2021 (2021). innovation, evidence, regulation, and adoption. Wooster, USA: IQVIA Institute. Available at: https://www.iqvia.com/insights/the-iqvia-institute/reports/digital-health-trends-2021 (accessed May 2021, 15).

[B7] Dong J. Shi G. H. Lu M. Huang S. Liu Y. H. Yao J. C. (2019). Evaluation of the predictive performance of Bayesian dosing for warfarin in Chinese patients. Pharmacogenomics 20 (3), 167–177. 10.2217/pgs-2018-0127 30777785

[B8] Hamberg A. K. Hellman J. Dahlberg J. Jonsson E. N. Wadelius M. (2015). A Bayesian decision support tool for efficient dose individualization of warfarin in adults and children. BMC Med. Inf. Decis. Mak. 15, 7. 10.1186/s12911-014-0128-0 PMC4324411 25889768

[B9] Hirsh J. Dalen J. E. Deykin D. Poller L. Bussey H. (1995). Oral anticoagulants. Mechanism of action, clinical effectiveness, and optimal therapeutic range. Chest 108 (4 Suppl. l), 231S-246S–246S. 10.1378/chest.108.4_supplement.231s 7555179

[B10] Holford N. H. (1986). Clinical pharmacokinetics and pharmacodynamics of warfarin. Understanding the dose-effect relationship. Clin. Pharmacokinet. 11 (6), 483–504. 10.2165/00003088-198611060-00005 3542339

[B11] Jiang J. Ji N. Lan J. Ge X. Du X. (2018). Clinical verification of Lou type warfarin pharmacokinetic dosing algorithms equation. Mol. Med. Rep. 17 (4), 6144–6149. 10.3892/mmr.2018.8562 29436624

[B12] Kearon C. Akl E. A. Ornelas J. Blaivas A. Jimenez D. Bounameaux H. (2016). Antithrombotic therapy for VTE disease: CHEST guideline and expert panel report. Chest 149 (2), 315–352. 10.1016/j.chest.2015.11.026 26867832

[B13] Lenzini P. Wadelius M. Kimmel S. Anderson J. L. Jorgensen A. L. Pirmohamed M. (2010). Integration of genetic, clinical, and INR data to refine warfarin dosing. Clin. Pharmacol. Ther. 87 (5), 572–578. 10.1038/clpt.2010.13 20375999 PMC2858245

[B14] Lin M. Q. Zhang J. Yu L. P. Song H. T. (2014). Advances of individualized administration model of warfarin based on pharmacogenomics. Chin. J. Clin. Pharmacol. Ther. [J] 19 (11), 1299–1305.

[B15] Liu W. F. Jia K. L. Xu C. R. Deng H. B. Chen J. (2021). Analysis of the guiding value of CYP2C9 and VKORC1 gene testing for individualized warfarin anticoagulant therapy in patients with acute pulmonary thromboembolism. J. Clin. Exp. Med. [J] 20 (15), 1618–1620. 10.3969/j.issn.1671-4695.2021.15.014

[B16] Marek E. Momper J. D. Hines R. N. Takao C. M. Gill J. C. Pravica V. (2016). Prediction of warfarin dose in pediatric patients: an evaluation of the predictive performance of several models. J. Pediatr. Pharmacol. Ther. 21 (3), 224–232. 10.5863/1551-6776-21.3.224 27453700 PMC4956330

[B17] Motykie G. D. Mokhtee D. Zebala L. P. Caprini J. A. Kudrna J. C. Mungall D. R. (1999). The use of a Bayesian forecasting model in the management of warfarin therapy after total hip arthroplasty. J. Arthroplasty 14 (8), 988–993. 10.1016/s0883-5403(99)90015-3 10614892

[B18] Schulman S. Angerås U. Bergqvist D. Eriksson B. Lassen M. R. Fisher W. (2010). Definition of major bleeding in clinical investigations of antihemostatic medicinal products in surgical patients. J. Thromb. Haemost. 8 (1), 202–204. 10.1111/j.1538-7836.2009.03678.x 19878532

[B19] Schulman S. Kearon C. Subcommittee on Control of Anticoagulation of the Scientific and Standardization Committee of the International Society on Thrombosis and Haemostasis (2005). Definition of major bleeding in clinical investigations of antihemostatic medicinal products in non-surgical patients. J. Thromb. Haemost. 3 (4), 692–694. 10.1111/j.1538-7836.2005.01204.x 15842354

[B20] Sennesael A. L. Larock A. S. Douxfils J. Elens L. Stillemans G. Wiesen M. (2018). Rivaroxaban plasma levels in patients admitted for bleeding events: insights from a prospective study. Thromb. J. 16, 28. 10.1186/s12959-018-0183-3 30455596 PMC6231259

[B21] Tan D. Yan H. Luo Z. Y. Liu R. Zhang W. Liu Z. Q. (2015). Research progress of establishing the gene-guided dosing predictive model of warfarin. Chin. J. Clin. Pharmacol. Ther. [J] 20 (12), 1434–1440.

[B22] Tavares L. C. Marcatto L. R. Santos P. C. J. L. (2018). Genotype-guided warfarin therapy: current status. Pharmacogenomics 19 (7), 667–685. 10.2217/pgs-2017-0207 29701078

[B23] Vahanian A. Beyersdorf F. Praz F. Milojevic M. Baldus S. Bauersachs J. (2022). 2021 ESC/EACTS Guidelines for the management of valvular heart disease. Eur. Heart J. 43 (7), 561–632. 10.1093/eurheartj/ehab395 34453165

[B24] Wright D. F. Duffull S. B. (2013). A Bayesian dose-individualization method for warfarin. Clin. Pharmacokinet. 52 (1), 59–68. 10.1007/s40262-012-0017-6 23329393

[B25] Zhang J. H. Liu M. B. Cai M. Z. (2022). Model-guided precision dosing of warfarin: a Chinese expert consensus (2022 edition). Chin. Clin. Pharmacol. Ther. 27 (11), 1201–1212.

